# Supplementary material for: Giant second harmonic transport under time-reversal symmetry in a trigonal superconductor
Source: Nat Commun. 2022 Mar 29;13:1659. doi: 10.1038/s41467-022-29314-4 (PMC8964720; doi:10.1038/s41467-022-29314-4)
Supplement: Supplementary file 1 — Supplementary Information [file 41467_2022_29314_MOESM1_ESM.pdf]

Supplementary Information for

Giant second harmonic transport under time−reversal symmetry in a trigonal superconductor

Supplementary Notes

1. Linear magnetotransport of $PbTaSe_2$

2. Vortex Hall effect probed by linear Hall effect in $PbTaSe_2$

3. Asymmetric vortex Hall effect as the origin of the nonlinear transport

4. Theoretical description of vortex-induced linear and nonlinear transport

5. Nonlinear anomalous transport in other samples

6. Nonlinear superconducting transport under the magnetic field

7. Comparison between nonlinear superconducting transport with and without magnetic field

8. Effect of Joule heating

9. Nonlinear transport in centrosymmetric superconductor

1. Linear magnetotransport of $PbTaSe_2$

In Supplementary Figs. 1a and 1b, we show the magnetic field dependence of longitudinal resistivity ρ_{xx} and transverse resistivity ρ_{yx}, respectively, at T = 8 K in sample 5. ρ_{xx} and ρ_{yx} are calculated by $\rho_{xx} = \frac{Wt}{L} R_{xx}^{\mathrm{sym}}$ and $\rho_{yx} = tR_{yx}^{\mathrm{asym}}$, respectively, where R_{xx}^{sym} (R_{yx}^{asym}) is the symmetrized longitudinal resistance (antisymmetrized transverse resistance) as a function of the magnetic field, W = 1.8 μA the width, L = 2.7 μA the length and t = 63 nm the thickness for sample 5. $\rho_{xx}(B)$ shows the simple positive magnetoresistance while $\rho_{yx}(B)$ shows the complex multi-carrier behavior. At the high field region, positive signal is dominant in $\rho_{yx}(B)$.

We analyzed $\rho_{yx}(B)$ by using the two-carrier model[1,2] (yellow dashed line in Supplementary Fig. 1b)

$$\rho_{yx}(B) = \frac{B}{e} \frac{\left(n_{\mathrm{h}}\mu_{\mathrm{h}}^2 - n_{\mathrm{e}}\mu_{\mathrm{e}}^2\right) + \mu_{\mathrm{h}}^2\mu_{\mathrm{e}}^2(n_{\mathrm{h}} - n_{\mathrm{e}})B^2}{(n_{\mathrm{h}}\mu_{\mathrm{h}} + n_{\mathrm{e}}\mu_{\mathrm{e}})^2 + \mu_{\mathrm{h}}^2\mu_{\mathrm{e}}^2(n_{\mathrm{h}} - n_{\mathrm{e}})^2B^2} \qquad (1)$$

where n_{h} (n_{e}) and μ_{h} (μ_{e}) are the carrier density and mobility of holes (electrons), respectively. Obtained fitting parameters are $n_{\mathrm{h}} = 5.7 \times 10^{22}$ cm^{-3}, $n_{\mathrm{e}} = 4.6 \times 10^{22}$ cm^{-3}, $\mu_{\mathrm{h}} = 2.1 \times 10^4$ cm^{2}V^{-1}s^{-1}, and $\mu_{\mathrm{e}} = 2.2 \times 10^4$ cm^{2}V^{-1}s^{-1}. Carrier density of hole is in the same order as one in 2H-$TaSe_2$ (3.5×10^{22} cm^{-3})[3], which implies that holes are mainly originating from $TaSe_2$ layers.

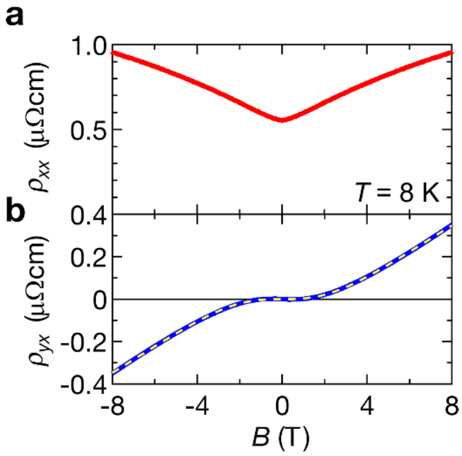

Supplementary Figure 1. Linear magnetotransport of $PbTaSe_2$. (a, b) Longitudinal resistivity ρ_{xx} (a) and transverse resistivity ρ_{yx} (b) at T = 8 K in sample 5. Yellow dashed line shows the fitting curve for $\rho_{yx}(B)$ by the two-carrier model.

2. Vortex Hall effect probed by linear Hall effect in $PbTaSe_2$

In the layered superconductors, the difference in chemical potentials in the normal core and the other superconducting region leads to the charging of vortices, which causes transverse motion of vortices and resultant anomalous Hall resistance near the superconducting transition[4–10]. Supplementary Figures 2a and 2b show the temperature dependence of symmetrized R_{xx}^{ω} (R_{xx}^{sym}, Supplementary Fig. 2a), antisymmetrized R_{xx}^{ω} (R_{xx}^{asym}, red) and R_{yx}^{ω} (R_{yx}^{asym}, blue) (Supplementary Fig. 2b) at $B = \pm 0.003$ T and $I = 10$ μA, respectively, for sample 5. During the superconducting transition, R_{yx}^{asym} shows the large negative peak. The sign of the Hall effect is opposite to R_{yx}^{asym} in the normal state, which is much smaller than the negative component as seen in Supplementary Fig. 1b, because we applied the magnetic field of only 0.003 T in order not to suppress superconductivity. Such a peak is absent in R_{xx}^{asym}. This anomalous sign reversal in the Hall resistance between the normal state and the superconducting state strongly suggests the occurrence of the vortex Hall effect in this layered superconductor. Supplementary Figures 2c and 2d display the magnetic field dependence of R_{xx}^{sym} and R_{yx}^{asym}, respectively, at $T = 3.1$ K (purple) and 3.4 K (orange) for sample 6. The current value was set at $I = 50$ μA in this measurement. The negative peak is clearly observed around the superconducting transition for both cases, which are consistent with the above scenario of the vortex Hall effect. We also calculated the Hall angle of vortex Hall effect as $r = \frac{R_{yx}^{\mathrm{asym}}(B)}{R_{xx}^{\mathrm{sym}}(B)} \frac{L}{W} = 0.005\text{-}0.01$ (see Supplementary Note 4 in detail), which is consistent with the other layered superconductors. Thus, we conclude that the Hall anomaly observed in $PbTaSe_2$ is attributed to the vortex Hall effect.

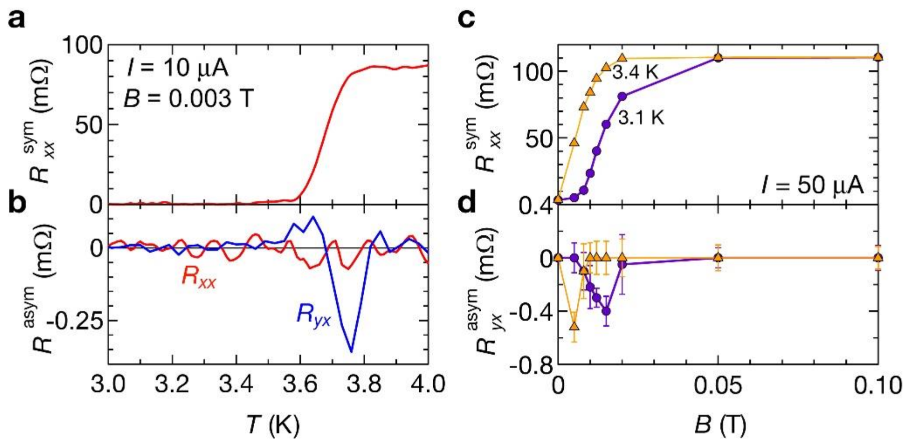

Supplementary Figure 2. Vortex Hall effect probed by linear Hall effect in $PbTaSe_2$. (a, b), Temperature dependence of R_{xx}^{sym} (a) and R^{asym} (b) at I = 10 μA and B = $\pm$0.003 T in sample 5. Red (blue) curve in Supplementary Fig. 2b shows R_{xx}^{asym} (R_{yx}^{asym}). (**c**, **d**) Magnetic field dependence of R_{xx}^{sym} (c) and R_{yx}^{asym} (d) at I = 50 μA and T = 3.1 K (purple), 3.4 K (orange) in sample 6. Errorbars indicate the uncertainty of the signals estimated from the temperature dependence of R_{xx}^{sym} under each magnetic field.

3. Asymmetric vortex Hall effect as the origin of the nonlinear transport

In Supplementary Figs. 3a and 3b, we propose a possible mechanism of the observed nonlinear transverse response (Supplementary Fig. 3a) and rectification effect (Supplementary Fig. 3b) in trigonal superconductors by considering the vortex/antivortex Hall effect on the trigonal potential. In the present samples of layered superconductor $PbTaSe_2$ of typically 80 nm in thickness, vortex/antivortex pairs are excited in the form of vortex string (Supplementary Fig. 3e) near the superconducting transition, as we explained later. We assumed that these excited vortex/antivortex pairs are subjected to the vortex Hall effect even in the absence of magnetic fields, where they are driven by their own magnetic flux in the same manner as in the presence of external magnetic field, discussed in the previous section (Supplementary Note 2). When the current is applied along the zigzag (armchair) direction, which corresponds to configuration A (B), vortices/antivortices are first driven to the armchair (zigzag) direction and then curved in the transverse zigzag (armchair) direction due to the vortex/antivortex Hall effect. The black/green arrows denote the motions of vortices/antivortices when the current flows leftward (rightward). During these processes, vortices/antivortices flows are rectified by the trigonal potential, which are drawn by the thickness differences of black and green arrows in Supplementary Figs. 3a and 3b. Thus, in configuration A (B), vortices/antivortices flow in parallel along the armchair (zigzag) direction and in antiparallel along the zigzag (armchair) direction. Parallel motion of vortices and antivortices cancel each other, whereas antiparallel motions of vortices/antivortices leads to the "vorticity current", which can be detected as the dc voltage in the direction perpendicular to those motions, causing the finite $R_{yx}^{2\omega}$ ($R_{xx}^{2\omega}$), or nonlinear transverse response (rectification effect) in configuration A (B). (We call this antiparallel motion "vorticity current" in analogy to the spin current in the inverse spin Hall effect. It is noted that the effect of vortices and antivortices driven in the same direction are canceled out.)

In above consideration, we assumed the existence of free vortex-antivortex in $PbTaSe_2$ in the absence of external magnetic field. This assumption is supported by the following model calculation based on Supplementary Ref. 12. In the layered superconductors, three types of excitations are known to occur[11,12]: (i) vortex-antivortex pair (Supplementary Fig. 3c), (ii) vortex ring (Supplementary Fig. 3d) and (iii) vortex string pair (Supplementary Fig. 3e). Vortex-antivortex pairs are vortex loops excited in each layer, which are independent from other ones in adjacent layers (Supplementary Fig. 3c). Vortex rings are vortex loops penetrating multiple layers within a sample (Supplementary Fig. 3d). Vortex string pairs are vortex loops penetrating all the layers (Supplementary Fig. 3e). Among these kinds of excitations, vortex string pairs are considered to be the origin of BKT-like transition in the layered superconductor $YBa_2Cu_3O_7$, in 10 layer-thick films[12]. Because the anisotropy parameter $\gamma = B_{\mathrm{c2}}^{\parallel}/B_{\mathrm{c2}}^{\perp}$ ($B_{\mathrm{c2}}^{\parallel}$ and $B_{\mathrm{c2}}^{\perp}$ are in-plane and out-of-plane critical magnetic field, respectively) of $YBa_2Cu_3O_7$ ($\gamma \lesssim 10$)[12] is in the same order as the one of $PbTaSe_2$ ($\gamma \sim 5$)[13], similar vortex excitations might exist at zero magnetic field in the present $PbTaSe_2$.

To evaluate the existence of the vortex string pairs in $PbTaSe_2$, we estimated the energies of vortex-antivortex pair (U_{vp}), vortex ring (U_{ring}) and vortex string pair (U_{str}). According to the previous studies[11,12], these energies are described as

$$U_{\mathrm{vp}} = K \ln\left(\frac{r_{\mathrm{v}}}{\xi_{ab}}\right) + K_{\perp}\left(\frac{r_{\mathrm{v}}}{d}\right)^2 + 2E_{\mathrm{c}} \quad (2)$$

$$U_{\mathrm{ring}} = \frac{r_{\mathrm{v}}}{d}\left[K \ln\left(\frac{r_{\mathrm{v}}}{\xi_{ab}}\right) + 2E_{\mathrm{c}}\right] \quad (3)$$

$$U_{\mathrm{str}} = n\left[K \ln\left(\frac{r_{\mathrm{v}}}{\xi_{ab}}\right) + 2E_{\mathrm{c}}\right] \quad (4)$$

where r_{v} is the diameter of vortex loop, K and $K_{\perp}$ are the intralayer and interlayer coupling constant, respectively, ξ_{ab} is the in-plane coherence length, d is the thickness of one layer, n is the number of layers and E_{c} is the energy to create one vortex in one layer. K and $K_{\perp}$ is

related to anisotropy parameter γ as $K/K_\perp = \gamma^2$ and we ignore E_c because it is small. Supplementary Figures 3f-h show U_vp (purple), U_ring (green) and U_str (blue) divided by K, as a function of r_v/ξ_{ab} at several temperatures. Here, we used $K/K_\perp = \gamma^2 = 25$, $\xi_{ab} = \xi_{ab0}\sqrt{\frac{T_c}{T_c - T}}$, where $\xi_{ab0} =$ 8 nm and T_c = 3.8 K, d = 1 nm and n = 100 in the present case of $PbTaSe_2$[13]. At T/T_c = 0 (Supplementary Fig. 3f), vortex ring has the lowest energy at small r_v region. However, at higher temperatures of T/T_c = 0.5 and 0.7 (Supplementary Figs. 3g and 3h), U_str decreases and becomes lower than U_ring, indicating that the vortex string pair is the lowest energy excitation in $PbTaSe_2$ above $T_\mathrm{c}/2$. This result is explained in terms of the enhancement of coherence length, that is, the vortex radius toward T_c, which makes the system more 2D-like. Thus, we conclude that vortex string pairs are excited in $PbTaSe_2$ flake near the superconducting transition and serve as free vortices and antivortices at zero magnetic field to cause the nonlinear superconducting transport.

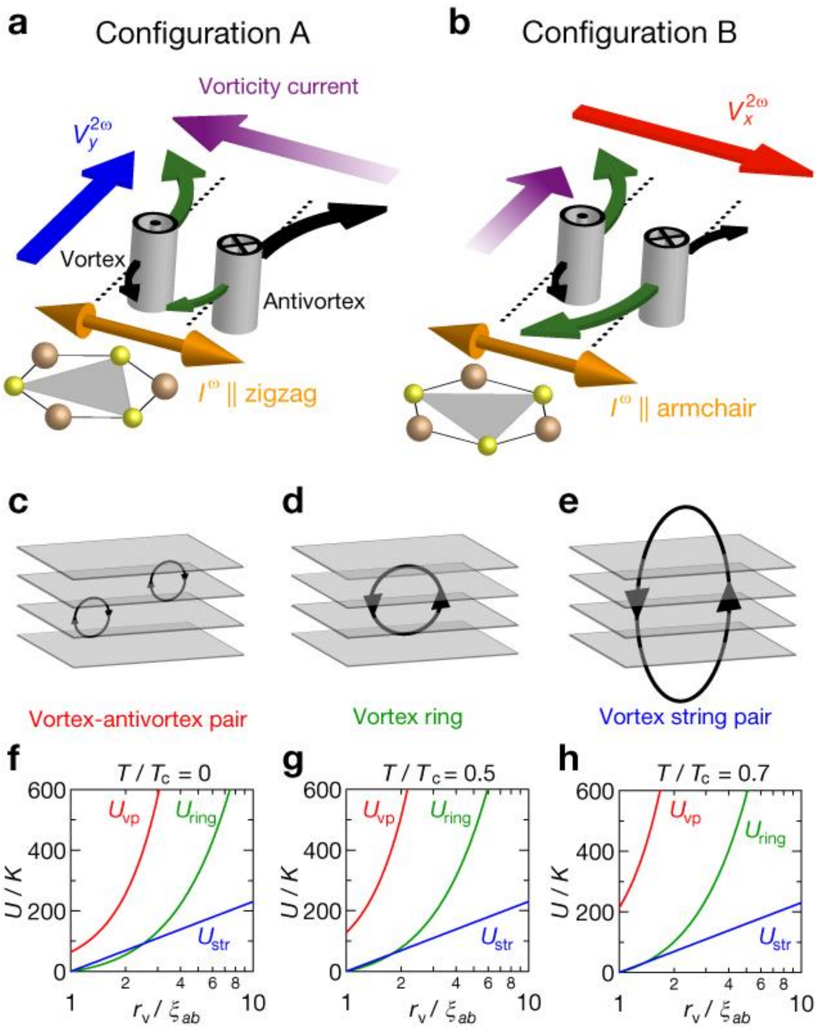

Supplementary Figure 3. Schematic images of asymmetric vortex Hall effect and vortex string pairs in $PbTaSe_2$. (**a**, **b**), Schematic images of rectified charged vortices/antivortices when the current is applied along zigzag (a) and armchair (b) directions. Black (green) arrows denote the trajectories of charged vortices/antivortices when the current flows leftward (rightward). The rectification of vortices/antivortices reflecting the trigonal potential is drawn by the thickness differences of arrows in the figures. Purple arrows show the antiparallel motions of vortices, or vorticity current. Nonlinear signals appear perpendicular to the vorticity current, in analogy to the inverse spin Hall effect. In configuration A (B), finite nonlinear transverse voltage $V_y^{2\omega}$ (nonlinear longitudinal voltage $V_x^{2\omega}$) is observed. (**c-e**), Schematic

images of vortex-antivortex pair (c), vortex ring (d) and vortex string pair (e) in the layered superconductor. (**f**-**h**), Excitation energy of vortex-antivortex pair (U_{vp}), vortex ring (U_{ring}) and vortex string pair (U_{str}) divided by intralayer coupling constant K at $T/T_c = 0$ (f), 0.5 (g) and 0.7 (h).

4. Theoretical description of vortex-induced linear and nonlinear transport

We describe the transport coefficients in terms of vortex contributions. With the weak force $\boldsymbol{F} = (F_x, F_y)$ acting on a vortex, the velocity for the vortex (vorticity $s = +$) or antivortex ($s = -$) is given by

$$\boldsymbol{v}^s = q_1 \begin{pmatrix} F_x \\ sF_y \end{pmatrix} + q_2 \begin{pmatrix} 2sF_xF_y \\ F_x^2 - F_y^2 \end{pmatrix} \quad (5)$$

The second term in the right-hand side originates from the trigonal symmetry with the configuration A. The motions of vortex and antivortex are switched by the mirror transformation with respect to the x axis. The voltage differences along x and y directions are given by the Josephson relation[14]

$$V_x = \phi_0^* L \sum_{s=\pm} sn^s v_y^s, \qquad V_y = \phi_0^* W \sum_{s=\pm} sn^s v_x^s \quad (6)$$

where $\phi_0^* = h/2|e|$ is the superconducting flux quantum, and n^s is the number density of vortices or antivortices. The sample lengths along x and y directions are L and W, respectively. Thus, the voltage is proportional to the vorticity current.

Next, we determine the concrete form of the force $\boldsymbol{F}$. For the linear response, the driving force $j\phi_0^*$ on a vortex, where j is the current density, is balanced by the force from environment as

$$sj\phi_0^*\hat{\boldsymbol{y}} = \eta_\parallel \boldsymbol{v}^s + s\eta_\perp \hat{\boldsymbol{z}} \times \boldsymbol{v}^s \quad (7)$$

where $\eta_\parallel$ is the friction coefficient and $\eta_\perp$ is responsible for the vortex Hall effect. The hat symbol represents a unit vector. Assuming that the Hall effect is small, i.e., $r \equiv \frac{\eta_\perp}{\eta_\parallel} \ll 1$, we obtain the relations $q_1 = \frac{1}{\eta_\parallel}$, $F_y = j\phi_0^*$, and $F_x = rF_y$.

As for the ratchet effect, we employ the information from the Brownian motion of point particle in the one-dimensional asymmetric potential for simplicity[15–17]. In order to determine the response coefficients, we focus on the case with the external field along the y-

direction ($F_x = 0, F_y \neq 0$). According to Supplementary Eq. (5), only the motion in the *y*-direction is involved, and then we consider the one-dimensional kinetic equation:

$$\eta_0 \partial_t X = -\partial_X U + F + \xi(t) \quad (8)$$

where X is the coordinate of the particle, ξ represents the force from a thermal noise which satisfies the random average $\langle \xi(t)\xi(t') \rangle = 2\eta_0 k_{\mathrm{B}} T \delta(t - t')$. The form of the potential is determined later. We define the positional distribution function $P(x,t) = \langle \delta(x - X(t)) \rangle$, which satisfies the Fokker-Planck equation

$$\eta_0 \partial_t P = \partial_x [(\partial_x U - F)P + k_{\mathrm{B}} T \partial_x P] \quad (9)$$

(See, for example, Appendix A5 of Supplementary Ref. 18 for a simple derivation). The solution of this equation can be found in Supplementary Refs 17 and 19. At the stationary condition $\partial_t P = 0$, we obtain the velocity

$$v = \langle \partial_t X \rangle = \int dx \ P(x) \frac{1}{\eta_0} (-\partial_x U + F) \quad (10)$$

as a function of the force. q_1 and q_2 are then obtained as the coefficients of F and F^2, respectively.

We take the potential form shown in Supplementary Fig. 4a, where ℓ_{v} is the periodicity of the potential for vortices, and the length $c\ell_{\mathrm{v}}$ is the size of each pinning center. The potential height is given by U, and we will take $c = 0.1$ in the numerical evaluation. From the Fokker-Planck equation, the response coefficients are given by

$$q_1 = \frac{1}{\eta_0} g_1(\beta U), \qquad q_2 = \frac{\beta \ell_{\mathrm{v}}}{\eta_0} g_2(\beta U), \quad (11)$$

$$g_1(x) = \frac{x^2}{D(x)}, \qquad D(x) = 2c^2(\cosh x - 1) + (1-c)^2 x^2 + 2(1-c)cx \sinh x,$$

$$g_2(x) = \frac{c^2 x}{D(x)^2} [4c + (2-c)x^2 - (4c - (1-c)x^2)\cosh x - (3-4c)x \sinh x], \quad (12)$$

where $\beta = \frac{1}{k_B T}$ is the inverse temperature. The form of Supplementary Eq. (11) itself can be deduced also from the dimensional analysis by recognizing that there exist the four dimensionful parameters η_0 [kg/s], ℓ_v [m], U_0 [J], $k_B T$[J]. While the actual potential shape is dependent on the system details, the coefficients $q_{1,2}$ generically have the exponential temperature dependence $e^{-\beta U}$ at low T reflecting the pinning of vortices. The constant η_0 is the friction coefficient without the pinning potentials. In the Bardeen-Stephen model[20], it is given by $\eta_0 = \frac{\pi \hbar^2 \sigma_n}{2e^2 \xi_{ab}^2}$ with the normal conductivity σ_n and the coherence length ξ_{ab}. While we have introduced the simplified ratchet potential phenomenologically by regarding the vortex as a point particle, it is difficult to derive the potential from the microscopic point of view. It may originate from the impurities and lattice defects, which pin the vortex in a collective manner for the weak pinning and the vortex may be pinned by a single center for the strong pinning. Our purpose here is to confirm the validity of the ratchet vortex scenario and hence we deal with the effective models introduced above. We also note that the randomness of the potential is neglected for simplicity, since the pinning effect and asymmetric feature, which are needed for the nonreciprocal transport, are accounted by our model.

1. Without magnetic field

First, we consider the case without magnetic field. In this case we have the relation $n^+ = n^- = n_v/2$ where n_v is the total number density of the vortices. We then obtain the linear and nonlinear transport coefficients as

$$R_{xx}^{\omega} = \frac{(\phi_0^*)^2 n_v L}{W \eta_0} g_1(\beta U), \qquad R_{xx}^{2\omega} = 0 \quad (13)$$

$$R_{yx}^{\omega} = 0, \qquad R_{yx}^{2\omega} = \frac{(\phi_0^*)^3 n_v r \beta \ell_v I}{W \eta_0} g_2(\beta U) \quad (14)$$

which satisfies the selection rule in the trigonal symmetry. Here we use the electrical current I instead of the current density. Note that the vortex Hall effect is essential for the nonreciprocal transport signal as seen from the presence of the factor $r = \eta_\perp/\eta_\parallel$. The ratio is written by the simple quantity

$$\frac{R_{yx}^{2\omega}}{R_{xx}^{\omega}} = \frac{\phi_0^* r \ell_{\mathrm{v}} I}{k_{\mathrm{B}} T L} \cdot \frac{g_2(\beta U)}{g_1(\beta U)} \quad (15)$$

which is not influenced by the number of vortices and friction coefficient. Taking the low-temperature limit, the expression is further simplified because $\frac{g_2}{g_1} = \frac{c}{2} = \mathrm{const.}$ can be used.

Let us evaluate the magnitude of the signals. First, we estimate the length ℓ_{v} from the information under the magnetic field at low temperature: At $B = B_{\mathrm{pin}}$, all the vortices with the number density B/ϕ_0^* are trapped by the pinning centers, and the mobile vortices appear for $B > B_{\mathrm{pin}}$ [17] which generate the transport signals (see Supplementary Fig. 7a in Supplementary Note 6). Hence the periodicity of pinning potential in the low-temperature regime is estimated as $\ell_{\mathrm{v0}} = \sqrt{\phi_0^*/B_{\mathrm{pin}}} \simeq 3 \times 10^{-7}$ m. On the other hand, the potential height U is estimated from the critical current I_{pin}, where the potential is well tilted by the external force and become flat at this critical current. Then we obtain the relation $U = \phi_0^* c \ell_{\mathrm{v}} I_{\mathrm{pin}}/W$. The value at low temperature is estimated by using $I_{\mathrm{pin,0}} \simeq 400\ \mu\mathrm{A}$ (see Supplementary Fig. 4b), and we get $U_0 \simeq 40$ meV. If we considered a temperature-independent potential height, the transport signal with the magnitude observed experimentally could not be reproduced due to the exponential factor $e^{-\beta U_0}$. Hence it is necessary and natural to consider the temperature dependence of U which goes to zero as T approaches to the mean-field critical temperature $T_{\mathrm{c}} \simeq 3.8$ K. In order to estimate the temperature dependence of potential height, we employ the condensation energy gain at the vortex core in the presence of the normal state at the impurity site as $U \simeq p \frac{B_{\mathrm{c}}^2}{2\mu_0} \pi \xi_{ab}^2 t \propto T_{\mathrm{c}} - T$ where B_{c} is the thermodynamic critical magnetic field, p

the fraction of pinning points[21,22], μ_0 the permeability in vacuum, and *t* the sample thickness. In addition, we assume the temperature dependence of ℓ_{v} as $\ell_{\mathrm{v}} \propto \sqrt{T_{\mathrm{c}} - T}$, which corresponds to the fact that the size of vortices increases as $T \rightarrow T_{\mathrm{c}}$ and the potential periodicity for vortices becomes effectively shorter together with the increasing coherence length ξ_{ab}. That is to say, the temperature-dependent variation of the vortex size, which cannot be treated in our model of Brownian motion of the point particle, was incorporated into ℓ_{v}. By considering the two general expressions of the pinning potential $U \propto c\ell_{\mathrm{v}} I_{\mathrm{pin}}$ (which was evaluated from the depinning process of vortices) and $U \propto T_{\mathrm{c}} - T$ (which was estimated from the energy gain of vortices) along with $\ell_{\mathrm{v}} \propto \sqrt{T_{\mathrm{c}} - T}$, we obtain $I_{\mathrm{pin}} \propto \sqrt{T_{\mathrm{c}} - T}$. Indeed, the experimental result supports this temperature dependence for the critical current (Supplementary Fig. 4b). In this way, we can write the temperature dependent parameters as $\ell_{\mathrm{v}}(T) \simeq \ell_{\mathrm{v}0}\sqrt{\frac{T_{\mathrm{c}}-T}{T_{\mathrm{c}}}}$ and $U(T) \simeq U_0 \frac{T_{\mathrm{c}}-T}{T_{\mathrm{c}}}$. Now we can evaluate the ratio $\frac{R_{xx}^{\omega}}{R_{yx}^{2\omega}}$. The temperature dependence near the transition point is shown in Supplementary Fig. 4c with the parameter $r = 0.01$ and the system lengths $L = 1.7$ µm, $W = 3.5$ µm. The magnitude is comparable to the experiments within the temperature range where the signal is observed.

Next, we consider the number of vortices which is necessary for the direct evaluation of $R_{yx}^{2\omega}$. We assume that the description of two-dimensional superconductors applies to the present system. Below the Kosterlitz-Thouless transition temperature T_{KT}, which is very close to the mean-field transition temperature T_{c} away from dirty limit[22,23], all the vortices are paired with antivortices. On the other hand, under the current flow, the mobile vortices are generated and the number of vortices is given at low current density by[14]

$$n_{\mathrm{v}}(I) = \frac{x}{2\pi\xi_{ab}^2}\left(\frac{I}{I_0}\right)^{2+\frac{x}{2}} \quad (16)$$

with $x = \frac{4(T_{\mathrm{KT}} - T)}{T_{\mathrm{KT}}}$ and $I_0 = \frac{k_{\mathrm{B}} T_{\mathrm{KT}} |e| W}{\hbar \xi_{ab}}$. If the current exceeds I_0, all the vortices are unpaired[24]. We can estimate I_0 by using the Ginzburg-Landau coherence length $\xi_{ab} = \xi_{ab0}\sqrt{\frac{T_{\mathrm{c}}}{T_{\mathrm{c}} - T}}$ with $\xi_{ab0} \simeq 40\ \mathrm{nm}$ [13], and we see that the experimentally used current $I = 100\ \mu\mathrm{A}$ exceeds this limit at all temperatures ($I/I_0(T) \gtrsim 10$). Hence, we assume that the existing vortices are free from vortex-antivortex binding. However, it is still not easy to estimate the number of vortices which could be generated by thermal fluctuations and current noise. Furthermore, the extrapolation of Supplementary Eq. (16) for the region $I > I_0$ does not work since the number of vortices much exceeds the upper bound $\sim \frac{1}{\pi \xi_{ab}^2}$ $(\sim n_{\mathrm{v}}(I = I_0))$. Then we consider the number density based on this maximum value and take $n_{\mathrm{v}} = \frac{\alpha'}{\pi \xi_{ab}^2}$ where $\alpha'(< 1)$ is the reduction factor from the maximum value. Here we use the current-independent expression for the number of vortices, which is motivated by the fact that the observed magnitudes at different currents does not strongly deviate as shown in Supplementary Fig. 4e.

The resistance itself can now be evaluated. Supplementary Figure 4d shows the temperature dependence of the nonlinear transverse signal $R_{yx}^{2\omega}$ for $\alpha' = 0.5$ and with the linear resistance $0.2\ \Omega$ in the normal state. The characteristic peak structure and the magnitude of the signal is consistent with the experimental observation. The absence of the signal in low-temperature region is not due to the vortex-antivortex binding but to the pinned vortices.

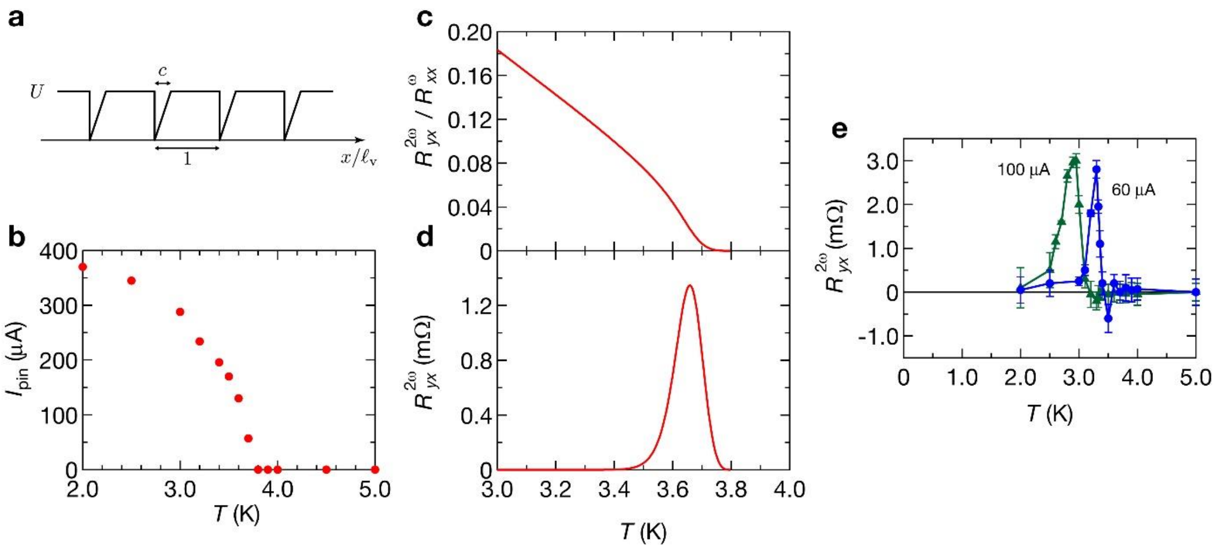

Supplementary Figure 4. Theoretically estimated nonlinear transport signals with configuration A. (**a**) Ratchet potential model used in this paper. (**b**) Temperature dependence of the critical current I_{pin} in sample 6. (**c**) Calculated ratio between nonlinear and linear transport signals near the transition temperature ($T_{\text{c}} \simeq T_{\text{KT}} \simeq 3.8$ K). (**d**) Calculated temperature dependence of the nonlinear transverse resistance. (**e**) Temperature dependence of transverse second harmonic resistance at different current. Blue circles and green triangles show R_{xx}^{ω} at I = 60 μA and 100 μA, respectively. Errorbars indicate the uncertainty of the signals estimated from the current dependence of $R_{yx}^{2\omega}$ at each temperature.

2. Comparison with signals in normal state

We compare the transport signals in the superconducting state with those of the normal state[25]. According to Supplementary Ref. 23, the linear and nonlinear transport signals in the normal state with time-reversal symmetry can be obtained from the Boltzmann transport theory as $j = \sigma_1 E + \sigma_2 E^2$, where

$$\sigma_1 \sim e n_e v_{\mathrm{F}} \frac{e\tau}{p_{\mathrm{F}}}, \qquad \sigma_2 \sim e n_e v_{\mathrm{F}} \left(\frac{e\tau}{p_{\mathrm{F}}}\right)^2 \frac{\tau}{\tilde{\tau}} \quad (17)$$

Here v_{F} is the Fermi velocity and p_{F} is the Fermi momentum, and then the Fermi energy is given by $\varepsilon_{\mathrm{F}} = \frac{1}{2} v_{\mathrm{F}} p_{\mathrm{F}}$. $n_e \sim k_{\mathrm{F}}^2$ is the number density of the normal electrons (k_{F} is the Fermi wavenumber). τ and $\tilde{\tau}$ are the scattering times for symmetric and skew scatterings, respectively. From these expressions we may evaluate the resistance ratio $R^{2\omega}/R^{\omega}$ for the normal state. As for the superconducting state, we have the ratio in Supplementary Eq. (15) and we take the low-temperature limit for the estimation of its magnitude. The ratio between normal and superconducting states is

$$\frac{(R^{2\omega}/R^{\omega})_{\mathrm{S}}}{(R^{2\omega}/R^{\omega})_{\mathrm{N}}} \sim \frac{\eta_{\perp}}{\eta_{\parallel}} \cdot \frac{\tilde{\tau}}{\tau} \cdot k_{\mathrm{F}} \ell_{\mathrm{v}} \cdot \frac{\varepsilon_{\mathrm{F}}}{k_{\mathrm{B}} T} \quad (18)$$

where $\eta_{\perp}/\eta_{\parallel}$ and $\tau/\tilde{\tau}$ are related to the Hall angles of the vortex and skew scattering of electron, respectively. Assuming that these factors are comparable, the difference in nonlinear transport signals is explained by the two huge factors $k_{\mathrm{F}} \ell_{\mathrm{v}}$ and $\varepsilon_{\mathrm{F}}/k_{\mathrm{B}} T$. Namely, the signal is much enhanced in the superconducting state because the nearly atomic length (k_{F}^{-1}) and Fermi energy are replaced by the much larger characteristic length for the vortices and the temperature, respectively, in the superconducting regime. This enhancement below superconducting transition temperature is consistent with the experimental observation discussed in Fig. 4 of the main text.

3. Under magnetic field

In the presence of the external magnetic field, the number of vortices is determined by the total magnetic flux. Then the number densities are given by $n^-(B) = 0$ and $n^+(B) = \frac{B}{\phi_0^*}$. Using Supplementary Eq. (6) we obtain the transport coefficients as

$$R_{xx}^{\omega}(B) = \frac{\phi_0^* BL}{\eta_0 W} g_1(\beta U), \qquad R_{xx}^{2\omega}(B) = \frac{(\phi_0^*)^2 B \ell_{\mathrm{v}} LI}{2\eta_0 k_{\mathrm{B}} T W^2} g_2(\beta U) \quad (19)$$

$$R_{yx}^{\omega}(B) = \frac{Wr}{L} R_{xx}^{\omega}(B), \qquad R_{yx}^{2\omega}(B) = \frac{2Wr}{L} R_{xx}^{2\omega}(B) \quad (20)$$

It is notable that the nonlinear Ohmic signal $R_{xx}^{2\omega}(B)$ is now finite and is not dependent on r originating from the vortex Hall effect. Here again the ratio between linear and nonlinear coefficients is given by the simple quantity

$$\frac{R_{xx}^{2\omega}(B)}{R_{xx}^{\omega}(B)} = \frac{\phi_0^* \ell_{\mathrm{v}} I}{2k_{\mathrm{B}} T W} \cdot \frac{g_2(\beta U)}{g_1(\beta U)} \quad (21)$$

Assuming that the properties of the pinning remain unchanged from the zero-field case, the ratio $R^{2\omega}(B)/R^{\omega}(B)$ in the magnetic field can be larger by the factor $r^{-1} = \eta_{\parallel}/\eta_{\perp}$ compared with Supplementary Eq. (15) for the zero-field case.

5. Nonlinear anomalous transport in other samples

Nonlinear anomalous transverse response and the rectification effect have been observed in other samples. In Supplementary Table 1, we summarized the nonlinear anomalous transport of all the samples we measured. In all samples, directional dependence of the nonlinear anomalous transport is consistent with the symmetry (configurations) and the magnitude of the nonlinear transport are in the same order.

As representatives, we show the current dependence of $R^{2\omega}$ of sample 1 (configuration A, $T = 20$ K) and sample 2 (configuration B, $T = 50$ K) in Supplementary Figs. 5a and 5b, and the current dependence of $R^{2\omega}$ (left) and R_{xx}^{ω} (right) of sample 6 (configuration A) and sample 7 (configuration B) at $T = 2$ K in Supplementary Figs. 5c and 5d. All the data of nonlinear anomalous transport ($R^{2\omega}$) show characteristic selection rules same as that mentioned in the main text (Figs. 2b, 2c, 3a and 3b) or in the previous section (Supplementary Note 3). In Supplementary Fig. 5e, we plotted $\frac{|E_y^{(2)}|}{\left(E_x^{(1)}\right)^2}$ vs. $(\sigma_{xx}^{\omega})^2$ in sample 1 (green squares) and sample 3 (pink triangles) with configuration A. In both samples, $\frac{|E_y^{(2)}|}{\left(E_x^{(1)}\right)^2}$ shows linear dependence on $(\sigma_{xx}^{\omega})^2$. Black and orange dotted lines show the fitting of $\frac{|E_y^{(2)}|}{\left(E_x^{(1)}\right)^2}$ in samples 1 and 3, respectively, by $\frac{|E_y^{(2)}|}{\left(E_x^{(1)}\right)^2} = \xi(\sigma_{xx}^{\omega})^2 + \eta$, where ξ and η are phenomenological fitting parameters. Here, ξ and η are estimated as $\xi = 2.3 \times 10^{-20}\ \mathrm{m^3V^{-1}\Omega^2}$ $(2.2 \times 10^{-20}\ \mathrm{m^3V^{-1}\Omega^2})$ and $\eta = -3.2\ \mathrm{\mu mV^{-1}}$, $(\eta = -30\ \mathrm{\mu mV^{-1}})$ for sample 1 (sample 3). It is noted that values of ξ in two samples are in similar order, implying that skew scattering induced nonlinear transverse response is also dominant in these samples.

Here, we show basic parameters of $PbTaSe_2$. We show temperature versus linear resistance R_{xx}^{ω} curves in samples 3, 4, 5, 6, and 7, which are not shown in the main text, in

Supplementary Figs. 6a-e. The values of residual resistivity ratio RRR = $R^{\omega}_{xx}(T = 300\ \mathrm{K})/R^{\omega}_{xx}(T = 2\ \mathrm{K})$ and transition temperature T_c are summarized in Supplementary Table 1. In Supplementary Fig. 6f, we plotted $\rho^{\omega}_{yx}(B)$ in sample 6 and analyzed it by using the two-carrier model (yellow dashed line) similarly to the analysis in the Supplementary Note 1. Obtained fitting parameters are $n_\mathrm{h} = 5.4 \times 10^{22}$ cm^{-3}, $n_\mathrm{e} = 4.8 \times 10^{22}$ cm^{-3}, $\mu_\mathrm{h} = 4.7 \times 10^{4}$ cm^{2}V^{-1}s^{-1}, and $\mu_\mathrm{e} = 5.0 \times 10^{4}$ cm^{2}V^{-1}s^{-1}. These values are in the same order as those in sample 5 shown in the Supplementary Note 1. We also note the characteristic parameters in superconducting state of $PbTaSe_2$: in-plane London length at T = 0 K is $\lambda_{ab}(0)$ = 82 nm and in-plane coherence length at T = 0 K is $\xi_{ab}(0)$ = 41 nm[13]. We estimated mean free path ℓ as follows. Because $PbTaSe_2$ is a semimetal with multi-carrier behavior and the main carrier is the hole, we roughly estimate ℓ as $\ell \sim \frac{\hbar k_\mathrm{F}}{n_h e^2 \rho^{\omega}_{xx}}$ ~ 69 nm, where k_F ~ 0.5 Å^{-1} is the Fermi wavenumber of the hole pocket[26], $n_\mathrm{h} = 5.7 \times 10^{22}$ cm^{-3} is the carrier density of holes in sample 5, $\rho^{\omega}_{xx} = 0.53$ μΩcm is the longitudinal resistivity in sample 5.

Sample No.	configuration	R^{ω}_{xx} (mΩ) (T = 5 K)	*RRR*	T_c (K)	$R^{2\omega}_{xx}$ (mΩ) (normal)	$R^{2\omega}_{yx}$ (mΩ) (normal)	$R^{2\omega}_{xx}$ (mΩ) (SC)	$R^{2\omega}_{yx}$ (mΩ) (SC)
1	A	200	14	3.09	0.09 (20 K)	**1.4 (20 K)**	0.76 (150 μA)	**2.4 (150 μA)**
2	B	48	95	3.61	**0.22 (50 K)**	-0.052 (50 K)	**1.0 (520 μA)**	-0.34 (520 μA)
3	A	47	40	3.66	0.1 (20 K)	**0.61 (20 K)**	-0.4 (170 μA)	**3.8 (170 μA)**
4	B	28	108	3.56	**1.4 (50 K)**	0.024 (50 K)	**1.1 (80 μA)**	-0.030 (80 μA)
5	B	87	79	3.68	**0.57 (20 K)**	-0.11 (20 K)	**4.8 (90 μA)**	1.8 (90 μA)
6	A	110	46	3.73	0.10 (20 K)	**0.22 (20 K)**	0.34 (310 μA)	**1.6 (310 μA)**
7	B	180	29	3.69	**0.52 (50 K)**	-0.05 (50 K)	**5.8 (270 μA)**	-0.78 (270 μA)

Supplementary Table 1. Summary of the nonlinear anomalous transport in all samples. $R^{2\omega}_{xx}$ and $R^{2\omega}_{yx}$ in the normal state is obtained at I = 3 mA. $R^{2\omega}_{xx}$ and $R^{2\omega}_{yx}$ in the superconducting state is estimated from the peak of $R^{2\omega}_{xx}$ ($R^{2\omega}_{yx}$) vs. I curves at T = 2 K.

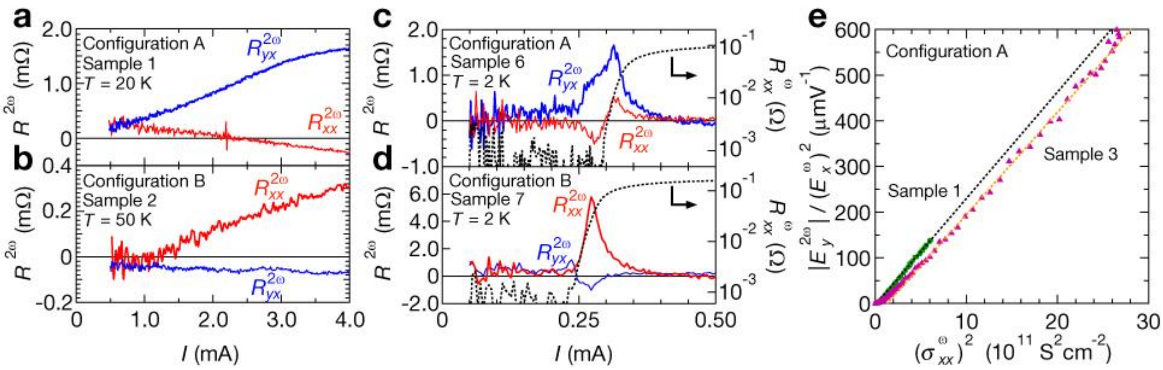

Supplementary Figure 5. Nonlinear anomalous transport under time-reversal symmetry in the normal state and superconducting state. (**a**, **b**), Current dependence of the second-harmonic resistance $R^{2\omega}$ in configuration A (sample 1) at T = 20 K (a) and configuration B (sample 2) at T = 50 K. (**c**, **d**), Current dependence of $R^{2\omega}$ (left) and R^{ω}_{xx} (right) at T = 2 K in configuration A (sample 6) (c) and configuration B (sample 7) (d). In Supplementary Figs. a-

d, red and blue lines indicate longitudinal ($R_{xx}^{2\omega}$) and transversal ($R_{yx}^{2\omega}$) resistance, respectively. (**e**), Normalized second-harmonic response $\frac{|E_y^{(2)}|}{\left(E_x^{(1)}\right)^2}$ as a function of $(\sigma_{xx}^{\omega})^2$ in sample 1 (green squares) and sample 3 (pink triangles). Sample 1 and 3 belong to configuration A. Black and orange dotted lines indicate the linear fitting in sample 1 and 3, respectively.

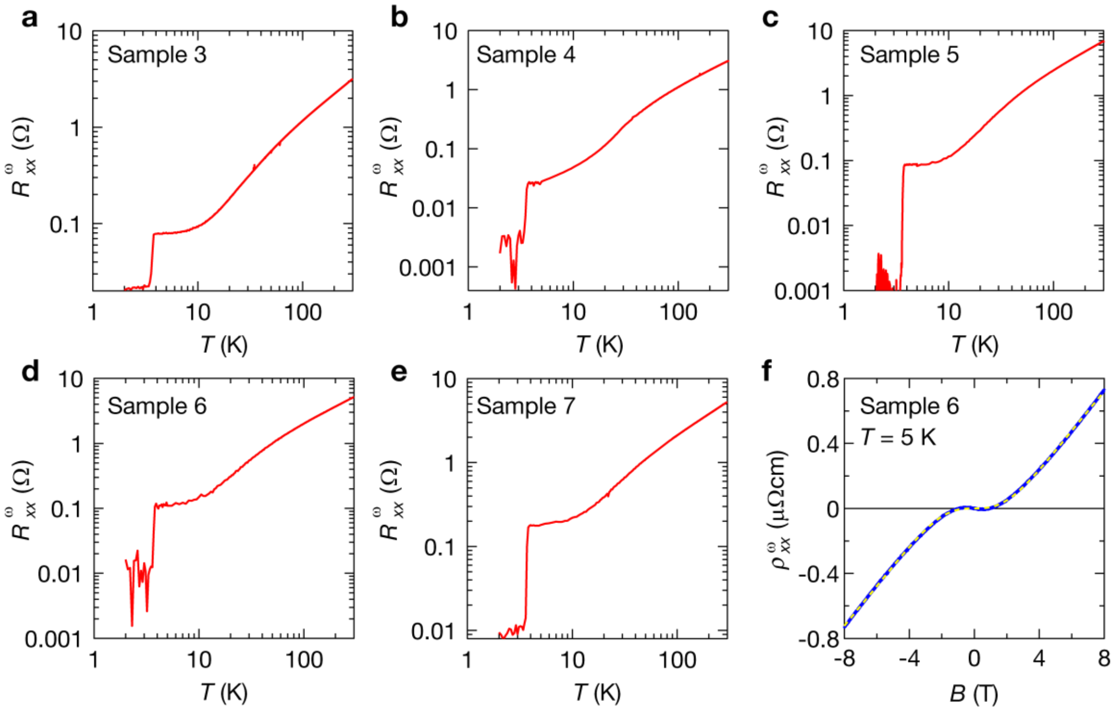

Supplementary Figure 6. Basic properties of measured samples. (**a-e**), Temperature dependences of the first harmonic resistance R_{xx}^{ω} in samples 3, 4, 5, 6, and 7. (**f**), Transverse resistivity ρ_{yx}^{ω} at $T = 5$ K in sample 6. Yellow dashed line shows the fitting curve for $\rho_{yx}^{\omega}(B)$ by the two-carrier model.

6. Nonlinear superconducting transport under the magnetic field

To achieve the comprehensive understanding of the nonlinear superconducting transport in trigonal $PbTaSe_2$, we measured the second-harmonic resistance under the magnetic field[27]. Supplementary Figures 7a and 7b show the magnetic field dependence of R_{xx}^{ω} (Supplementary Fig. 7a), $R_{xx}^{2\omega}$ (red) and $R_{yx}^{2\omega}$ (blue) (Supplementary Fig. 7b), in sample 6. In this measurement, the current flows along the zigzag direction (configuration A). We observed clear peak structure in $R_{xx}^{2\omega}$ during the superconducting transition, whose sign is reversed by inverting the magnetic field. Such signals are indiscernible in $R_{yx}^{2\omega}$. Such behavior of nonlinear transport under the magnetic field is called nonreciprocal magnetotransport (or magnetochiral anisotropy), which indicates the rectification effect under broken time-reversal symmetry, and is consistent with previous studies[27,28]. It is important to note that the directional dependence of the second-harmonic signals (i.e., directions along which nonlinear transport appear) are rotated by 90 degree between the nonlinear anomalous transport under time-reversal symmetric condition and nonreciprocal magnetotransport, which are all consistent with the symmetry argument. These results strongly suggest the intrinsic nature of the observed signals. In Supplementary Figs. 7c and 7d, we also show the temperature dependence of R_{xx}^{ω} (Supplementary Fig. 7c), $R_{xx}^{2\omega}$ (red) and $R_{yx}^{2\omega}$ (blue) (Supplementary Fig. 7d) in sample 6. As discussed in the main text, we observe the large peak in $R_{yx}^{2\omega}$ during the superconducting transition and negligible signals in $R_{xx}^{2\omega}$.

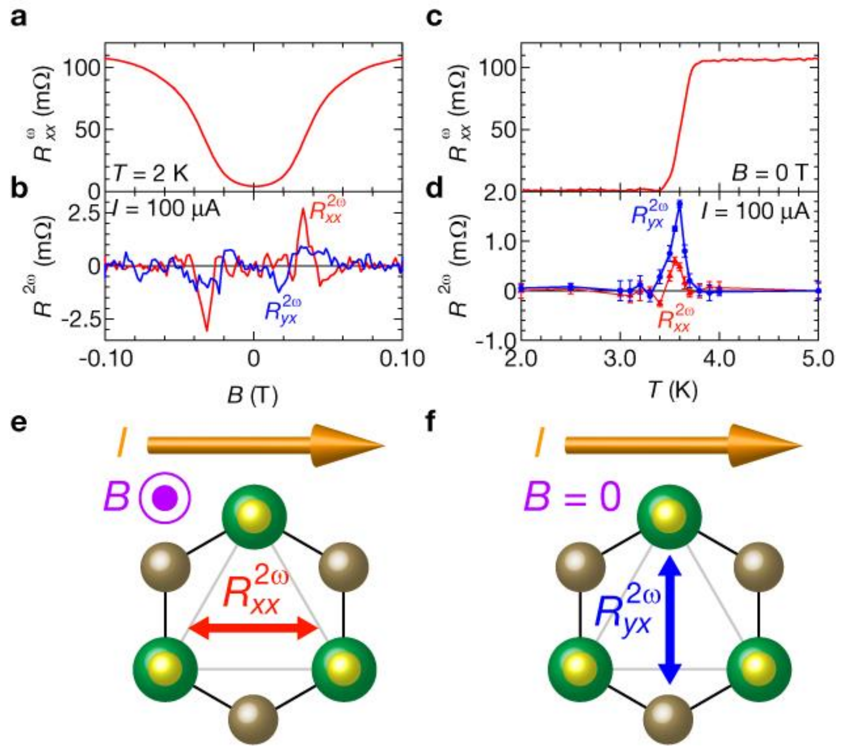

Supplementary Figure 7. Nonreciprocal magnetotransport in $PbTaSe_2$. (**a**, **b**) Magnetic field dependence of the first harmonic longitudinal resistance (R^{ω}_{xx}) and second-harmonic resistance ($R^{2\omega}$) in the longitudinal ($R^{2\omega}_{xx}$) and transverse ($R^{2\omega}_{yx}$) directions at T = 2 K and I = 100 μA in sample 6. Red (blue) curve in Supplementary Fig. b shows $R^{2\omega}_{xx}$ ($R^{2\omega}_{yx}$). (**c, d**) Temperature dependence of the first harmonic longitudinal resistance (R^{ω}_{xx}) and second-harmonic resistance ($R^{2\omega}$) in the longitudinal ($R^{2\omega}_{xx}$) and transverse ($R^{2\omega}_{yx}$) directions at B = 0 T and I = 100 μA in sample 6. Red (blue) curve in Supplementary Fig. d shows $R^{2\omega}_{xx}$ ($R^{2\omega}_{yx}$). When the magnetic field (~ 0.033 T) is applied, $R^{2\omega}_{xx}$ shows peak structure (nonreciprocal magnetoresistance) while $R^{2\omega}_{yx}$ is dominant (nonlinear transverse response discussed in the main text) under the zero magnetic field in the superconducting state. Errorbars indicate the uncertainty of the signals estimated from the current dependence of $R^{2\omega}$ at each temperature. (**e, f**) Schematic image of the nonlinear transport in configuration A under out-of-plane magnetic field (e) and without magnetic field (f). Under (without) magnetic field, the second-harmonic signal is expected in the longitudinal (transverse) direction.

7. Comparison between nonlinear superconducting transport with and without magnetic field

We now compare the magnitude of nonlinear transport with and without magnetic field. By combining Supplementary Eqs. (13), (15), (19), and (21), we can obtain the ratio between $R_{yx}^{2\omega}$ and $R_{xx}^{2\omega}(B)/B$, where $R_{yx}^{2\omega}$ and $R_{xx}^{2\omega}(B)$ are nonlinear transverse resistance without magnetic field and nonlinear longitudinal resistance under magnetic field, respectively, as

$$\frac{R_{yx}^{2\omega}}{R_{xx}^{2\omega}(B)/B} = \frac{2W}{L}\phi_0^* n_{\mathrm{v}} r. \quad (22)$$

To estimate the *r* value from nonlinear transport measurements, we employed the experimental values at $I = 200$ μA in sample 6. Supplementary Fig. 8a shows the current dependence of $R_{yx}^{2\omega}$ at $T = 2$ K and $B = 0$ T. The experimental value of $R_{yx}^{2\omega} = 0.20$ mΩ at $I = 200$ μA is extracted as shown by black dashed line in Supplementary Fig. 8a. Supplementary Fig. 8b shows the magnetic field dependence of $R_{xx}^{2\omega}$ at $T = 2$ K and $I = 200$ μA. By using the peak amplitude of $R_{xx}^{2\omega}(B) = 0.91$ mΩ and the magnetic field at the peak position = 0.0089 T as an approximation, we estimated the experimental value of $R_{xx}^{2\omega}(B)/B = 0.10$ Ω/T. The slope of $R_{xx}^{2\omega}(B)$ as a function of *B* is shown by gray dashed line in Supplementary Fig. 8b. By combining the values obtained above and other parameters, we calculated $r \sim 0.0025$, which is close to the value obtained from linear transport ($r = 0.005$-0.01) as shown in Supplementary Note 2.

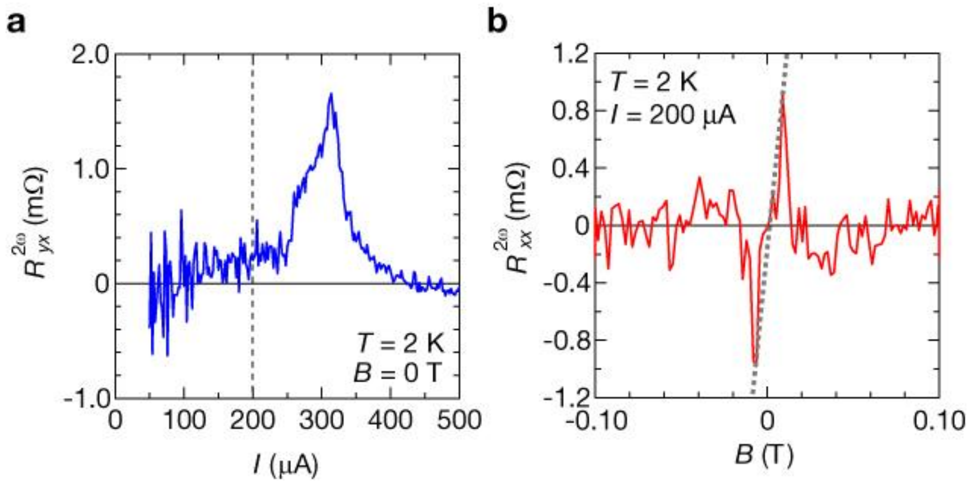

Supplementary Figure 8. Comparison between nonlinear transport with and without magnetic field. (**a**) Current dependence of $R_{yx}^{2\omega}$ at T = 2 K and B = 0 T in sample 6. Black dashed line means I = 200 μA, where we estimated the value of r. (**b**) Magnetic field dependence of $R_{xx}^{2\omega}$ at T = 2 K and I = 200 μA in sample 6. Gray dashed line indicates the slope of $R_{xx}^{2\omega}(B)$ as a function of B, which is estimated by using the peak amplitude of $R_{xx}^{2\omega}(B)$ and the magnetic field at the peak position.

8. Effect of Joule heating

We consider the heating effect on the transport coefficients caused by the external current[29,30]. The current-voltage relation is in general written as

$$V = R^{(1)}(T)I + R^{(2)}(T)I^2 + R^{(3)}(T)I^3 + \cdots \quad (23)$$

where T is the temperature of the sample. The Joule heating is accounted by the power $P = VI$. The energy transfer from the sample to the environment with the temperature T_0 is given by $P = G\delta T$ where G is the thermal boundary conductance and $\delta T = T - T_0$ represents the temperature variation. Then, the temperature change δT is expressed as a function of the current, and the leading-order term is proportional to the square of the current. We thereby obtain

$$V = R^{(1)}(T_0)I + R^{(2)}(T_0)I^2 + \left[R^{(3)}(T_0) + \frac{R^{(1)}(T_0)R^{(1)'}(T_0)}{G(T_0)}\right] I^3 + \cdots \quad (24)$$

where $R^{(1)'} = \partial_T R^{(1)}$ is the temperature derivative of the resistance. It is notable that there is a qualitative difference between the second-order and third-order terms. Namely, the third-order contribution can be generated through the combination of the first-order resistance and the temperature-varying effect, but the second-order coefficient is not generated by such an effect. Hence the second-order signals studied in this work captures only the intrinsic effect from the nonreciprocal response in the noncentrosymmetric superconductors.

9. Nonlinear transport in centrosymmetric superconductor

In order to further clarify that second harmonic response comes from the noncentrosymmetric crystal symmetry, we investigated the centrosymmetric superconductor as a control experiment. As a comparable centrosymmetric superconductor, we investigated the second-order nonlinear response in 2H-$NbSe_2$. In $PbTaSe_2$ (Supplementary Fig. 9a), all the $TaSe_2$ layers are stacked in the same direction, leading to the noncentrosymmetric crystal structure (space group of $P\bar{6}/m2$) as shown in the main text. With contrast, in 2H-$NbSe_2$ (Supplementary Fig. 9b), neighboring $NbSe_2$ layers are rotated by 180°. Thus, bulk 2H-$NbSe_2$ becomes centrosymmetric (space group of $P6_3/mmc$). We prepared 2H-$NbSe_2$ device (thickness t = 103 nm) in configuration A (current flowing along the zigzag direction as shown in Supplementary Fig. 9c) and measured the first and second harmonic resistance similarly to $PbTaSe_2$ (Fig. 3a in the main text). 2H-$NbSe_2$ shows metallic behavior and superconductivity around T = 7 K (Supplementary Fig. 9d), which is consistent with previous studies[31,32].

First, we focus on the nonlinear signals in the normal state. Supplementary Figure 10a (b) shows $R_{xx}^{2\omega}$ and $R_{yx}^{2\omega}$ as a function of the current in $PbTaSe_2$ (2H-$NbSe_2$) at T = 20 K. $PbTaSe_2$ shows the large second harmonic signal in $R_{yx}^{2\omega}$ while $R_{xx}^{2\omega}$ is small (nonlinear transverse response). With contrast, in 2H-$NbSe_2$ both $R_{xx}^{2\omega}$ and $R_{yx}^{2\omega}$ are indicernible. This indicates that the intrinsic second harmonic signal is absent in centrosymmetric system.

Next, we discuss the nonlinear signals in the superconducting state. Supplementary Figure 10c (d) shows the current dependence of $R_{xx}^{2\omega}$ and $R_{yx}^{2\omega}$ in $PbTaSe_2$ (2H-$NbSe_2$) at T = 2 K. $R_{yx}^{2\omega}$ shows the peak sturucture during the superconducting transition while the signal in $R_{xx}^{2\omega}$ is indiscernible as discussed in the main text. With contrast, both $R_{xx}^{2\omega}$ and $R_{yx}^{2\omega}$ in 2H-$NbSe_2$ are negligibly small, which is the expected behavior of the centrosymmetric crystals. We note a small peak structure in $R_{xx}^{2\omega}$ in the transition, which also appears in $R_{xx}^{2\omega}$ of $PbTaSe_2$. This might come from the asymmetry of the contacts or inhomogeneiety of the

superconductivity. However, its magnitude is much smaller than the intrinsic signals of the nonlinear transverse response in $PbTaSe_2$ (Supplementary Fig. 10c).

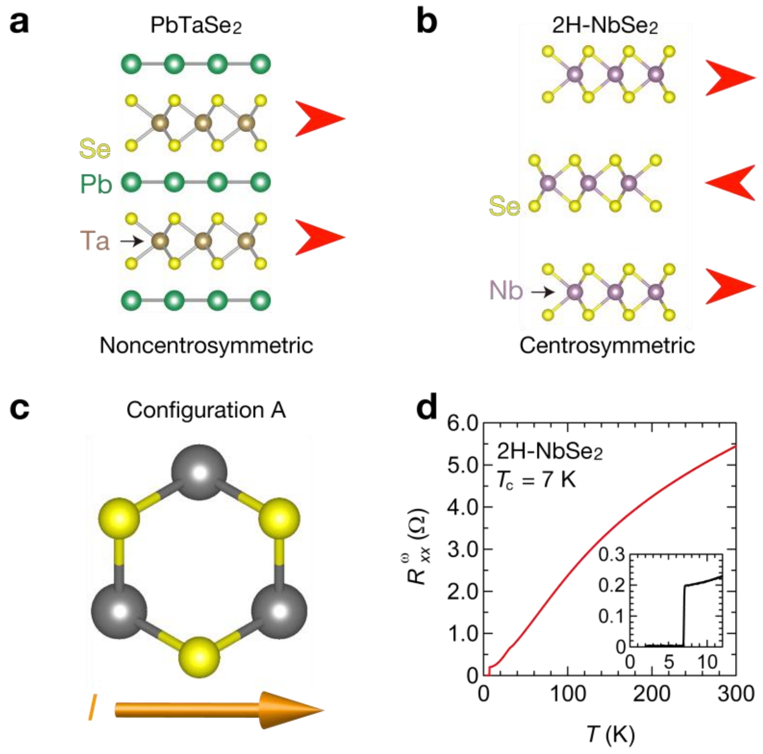

Supplementary Figure 9. Schematic crystal structures of noncentrosymmetric $PbTaSe_2$ and centrosymmetric 2H-$NbSe_2$, and superconducting property in 2H-$NbSe_2$. (**a**, **b**) Schematic crystal structure of $PbTaSe_2$ (a) and 2H-$NbSe_2$ (b). In $PbTaSe_2$, all $TaSe_2$ layers are stacked in the same direction, leading to the noncentrosymmetric crystal structure. On the other hand, in 2H-$NbSe_2$, neighboring $NbSe_2$ layers are rotated by 180°, resulting in the centrosymmetric crystal structure. Red wedges indicate the direction of each $TaSe_2$ ($NbSe_2$) layers. (**c**), Schematic image of the applied current along zigzag direction in $TaSe_2$ ($NbSe_2$) layer. (**d**), Temperature dependence of R_{xx}^{ω} in 2H-$NbSe_2$ when $I = 50$ μA. The inset shows R_{xx}^{ω} around the superconducting transition ($T_c = 7$ K).

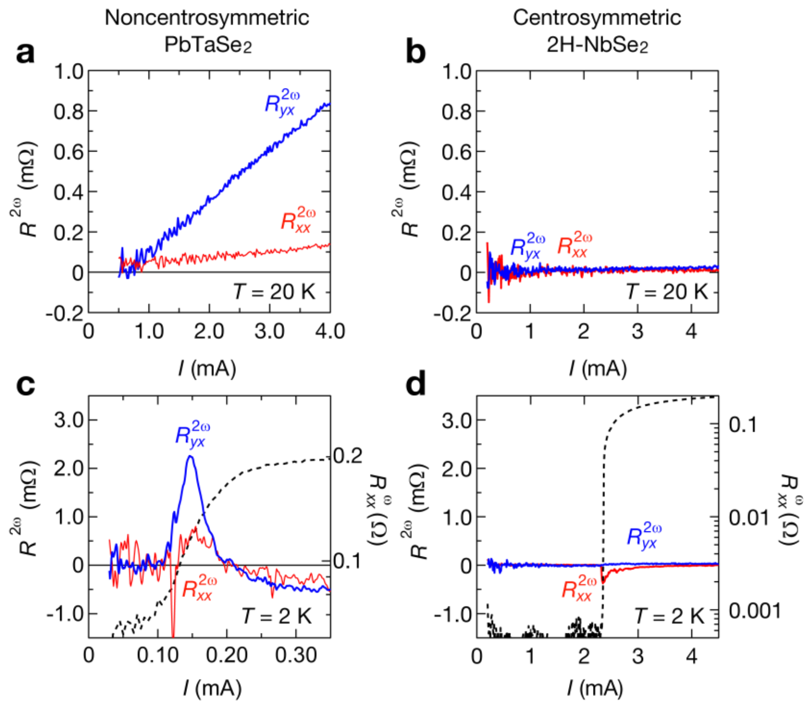

Supplementary Figure 10. Comparison between noncentrosymmetric $PbTaSe_2$ and centrosymmetric 2H-$NbSe_2$. (**a**, **b**) Current dependences of the second harmonic resistance $R^{2\omega}$ in $PbTaSe_2$ (a) and 2H-$NbSe_2$ (b) at $T = 20$ K. (**c**, **d**), Current dependence of $R^{2\omega}$ (left) and R^{ω}_{xx} (right) at $T = 2$ K in $PbTaSe_2$ (c) and 2H-$NbSe_2$ (d). In Supplementary Figs. a-d, red and blue lines indicate longitudinal ($R^{2\omega}_{xx}$) and transverse ($R^{2\omega}_{yx}$) resistance, respectively.

Supplementary References

1. Li, C. Z. *et al.* Two-carrier transport induced Hall anomaly and large tunable magnetoresistance in Dirac semimetal Cd_3As_2 nanoplates. *ACS Nano* **10**, 6020–6028 (2016).
2. Rourke, P. M. C. *et al.* Fermi-surface reconstruction and two-carrier model for the Hall effect in $YBa_2Cu_4O_8$. *Phys. Rev. B* **82**, 020514 (2010).
3. Naito, M. & Tanaka, S. Electrical transport properties in 2H-NbS_2, -$NbSe_2$, -TaS_2 and -$TaSe_2$. *J. Phys. Soc. Japan* **51**, 219–227 (1982).
4. Hagen, S. J., Lobb, C. J., Greene, R. L., Forrester, M. G. & Kang, J. H. Anomalous Hall effect in superconductors near their critical temperatures. *Phys. Rev. B* **41**, 11630–11633 (1990).
5. Luo, J., Orlando, T. P., Graybeal, J. M., Wu, X. D. & Muenchausen, R. Scaling of the longitudinal and Hall resistivities from vortex motion in $YBa_2Cu_3O_7$. *Phys. Rev. Lett.* **68**, 690–693 (1992).
6. Hagen, S. J. *et al.* Anomalous flux-flow Hall effect: $Nd_{1.85}Ce_{0.15}CuO_{4-y}$ and evidence for vortex dynamics. *Phys. Rev. B* **47**, 1064–1068 (1993).
7. Khomskii, D. I. & Freimuth, A. Charged vortices in high temperature superconductors. *Phys. Rev. Lett.* **75**, 1384–1386 (1995).
8. Kopnin, N. B. & Lopatin, A. V. Flux-flow Hall effect in clean type-II superconductors. *Phys. Rev. B* **51**, 15291–15303 (1995).
9. Nagaoka, T. *et al.* Hall anomaly in the superconducting state of high-T_c cuprates: universality in doping dependence. *Phys. Rev. Lett.* **80**, 3594–3597 (1998).
10. Auerbach, A. & Arovas, D. P. Hall anomaly and moving vortex charge in layered superconductors. *SciPost Phys.* **8**, 061 (2020).
11. Hikami, S. & Tsuneto, T. Phase Transition of Quasi-Two Dimensional Planar System.

Prog. Theor. Phys. **63**, 387–401 (1980).

12. Matsuda, Y. *et al.* Thickness dependence of the Kosterlitz-Thouless transition in ultrathin $YBa_2Cu_3O_{7-\delta}$ films. *Phys. Rev. B* **48**, 10498 (1993).
13. Namiki, H. & Sasagawa, T. Anisotropic superconducting properties of noncentrosymmetric $PbTaSe_2$ as a candidate exotic superconductor. *Sci. Adv. Mater.* **8**, 2097–2102 (2016).
14. Halperin, B. I. & Nelson, D. R. Resistive transition in superconducting films. **36**, 599–616 (1979).
15. Ambegaokar, V., Halperin, B. I., Nelson, D. I. & Siggia, E. D. Dynamics of superfluid films. *Phys. Rev. B* **21**, 1806–1826 (1980).
16. Zhu, Y., Marchesoni, F., Moshchalkov, V. & Nori, F. Controllable step motors and rectifiers of magnetic flux quanta using periodic arrays of asymmetric pinning defects. *Phys. Rev. B* **68**, 014514 (2003).
17. Hoshino, S., Wakatsuki, R., Hamamoto, K. & Nagaosa, N. Nonreciprocal charge transport in two-dimensional noncentrosymmetric superconductors. *Phys. Rev. B* **98**, 054510 (2018).
18. Risken, H. *The Fokker-Planck equation (Second Edition)*, (Springer, 1989).
19. Reimann, P. *et al.* Giant acceleration of free diffusion by use of tilted periodic potentials. *Phys. Rev. Lett.* **87**, 010602 (2001).
20. Bardeen, J. & Stephen, M. J. Theory of the motion of vortices in superconductors. *Phys. Rev.* **140**, (1965).
21. Anderson, P. W. & Kim, Y. B. Hard superconductivity: Theory of the motion of Abrikosov flux lines. *Rev. Mod. Phys.* **36**, 39–43 (1964).
22. Hsu, J. W. P. & Kapitulnik, A. Superconducting transition, fluctuation, and vortex motion in a two-dimensional single-crystal Nb film. *Phys. Rev. B* **45**, 4819–4835

(1992).

23. Beasley, M. R., Mooij, J. E. & Orlando, T. P. Possibility of vortex-antivortex pair dissociation in two-dimensional superconductors. *Phys. Rev. Lett.* **42**, 1165–1168 (1979).

24. McCauley, J. L. Dissociation of a two-dimensional Coulomb gas at low temperatures. *J. Phys. C Solid State Phys.* **10**, 689–692 (1977).

25. Isobe, H., Xu, S. Y. & Fu, L. High-frequency rectification via chiral Bloch electrons. *Sci. Adv.* **6**, eaay2497 (2020).

26. Bian, G. *et al.* Topological nodal-line fermions in spin-orbit metal $PbTaSe_2$. *Nat. Commun.* **7**, 10556 (2016).

27. Ideue, T., Koshikawa, S., Namiki, H., Sasagawa, T. & Iwasa, Y. Giant nonreciprocal magnetotransport in bulk trigonal superconductor $PbTaSe_2$. *Phys. Rev. Res.* **2**, 042046 (2020).

28. Itahashi, Y. M., Saito, Y., Ideue, T., Nojima, T. & Iwasa, Y. Quantum and classical ratchet motions of vortices in a two-dimensional trigonal superconductor. *Phys. Rev. Res.* **2**, 023127 (2020).

29. Mishonov, T. M., Chéenne, N., Robbes, D. & Indekeu, J. O. Generation of 3rd and 5th harmonics in a thin superconducting film by temperature oscillations and isothermal nonlinear current response. *Eur. Phys. J. B* **26**, 291–296 (2002).

30. Ossandón, J. G. *et al.* Non-linear response of ac conductivity in narrow YBCO film strips at the superconducting transition. *J. Phys. Conf. Ser.* **43**, 655–658 (2006).

31. Foner, S. & McNiff, E. J. Upper critical fields of layered superconducting $NbSe_2$ at low temperature. *Phys. Lett. A* **45**, 429–430 (1973).

32. Xi, X. *et al.* Ising pairing in superconducting $NbSe_2$ atomic layers. *Nat. Phys.* **12**, 139–143 (2016).
